# Malnutrition outweighs the effect of the obesity paradox

**DOI:** 10.1002/jcsm.12980

**Published:** 2022-03-29

**Authors:** Suriya Prausmüller, Gregor Heitzinger, Noemi Pavo, Georg Spinka, Georg Goliasch, Henrike Arfsten, Cornelia Gabler, Guido Strunk, Christian Hengstenberg, Martin Hülsmann, Philipp E. Bartko

**Affiliations:** ^1^ Division of Cardiology, Department of Internal Medicine, II Medical University of Vienna Vienna Austria; ^2^ IT Systems and Communications Medical University of Vienna Vienna Austria; ^3^ Complexity Research Vienna Austria

**Keywords:** Malnutrition, Obesity paradox, Heart failure, PNI

## Abstract

**Background:**

High body mass index (BMI) is paradoxically associated with better outcome in patients with heart failure (HF). The effects of malnutrition on this phenomenon across the whole spectrum of HF have not yet been studied.

**Methods:**

In this observational study, patients were classified by guideline diagnostic criteria to one of three heart failure subtypes: reduced (HFrEF), mildy reduced (HFmrEF), and preserved ejection fraction (HFpEF). Data were retrieved from the Viennese‐community healthcare provider network between 2010 and 2020. The relationship between BMI, nutritional status reflected by the prognostic nutritional index (PNI), and survival was assessed. Patients were classified by the presence (PNI < 45) or absence (PNI ≥ 45) of malnutrition.

**Results:**

Of the 11 995 patients enrolled, 6916 (58%) were classified as HFpEF, 2809 (23%) HFmrEF, and 2270 HFrEF (19%). Median age was 70 years (IQR 61–77), and 67% of patients were men. During a median follow‐up time of 44 months (IQR 19–76), 3718 (31%) of patients died. After adjustment for potential confounders, BMI per IQR increase was independently associated with better survival (adj. hazard ratio [HR]: 0.91 [CI 0.86–0.97], *P* = 0.005), this association remained significant after additional adjustment for HF type (adj. HR: 0.92 [CI 0.86–0.98], *P* = 0.011). PNI was available in 10 005 patients and lowest in HFrEF patients. PNI was independently associated with improved survival (adj. HR: 0.96 [CI 0.95–0.97], *P* < 0.001); additional adjustment for HF type yielded similar results (adj. HR: 0.96 [CI 0.96–0.97], *P* < 0.001). Although obese patients experienced a 30% risk reduction, malnutrition at least doubled the risk for death with 1.8‐ to 2.5‐fold higher hazards for patients with poor nutritional status compared with normal weight well‐nourished patients.

**Conclusions:**

The obesity paradox seems to be an inherent characteristic of HF regardless of phenotype and nutritional status. Yet malnutrition significantly changes trajectory of outcome with regard to BMI alone: obese patients with malnutrition have a considerably worse outcome compared with their well‐nourished counterparts, outweighing protective effects of high BMI alone. In this context, routine recommendation towards weight loss in patients with obesity and HF should generally be made with caution and focus should be shifted on nutritional status.

## Introduction

Obesity as a well‐established risk factor is known to greatly increase risk for the development of cardiovascular disease including heart failure (HF), setting obesity as the world's leading preventable risk factor for early death.^1^ Paradoxically, once a patient develops HF, high body mass index (BMI) seems to confer a survival advantage compared with leaner individuals, a phenomenon commonly referred to as the ‘obesity paradox’.^2^ Several explanations have been put forth to explain this association. To date, it is not entirely clear whether this is a true phenomenon or a consequence of methodological limitations such as confounding or reverse causation. Earlier appearance of symptoms in obese individuals at the one hand and disease‐associated weight loss, smoking status, and muscle wasting, on the other, have been identified as possible reasons for the obesity paradox.^3^, ^4^

Although often ignored, malnutrition is highly prevalent among patients with HF and is associated with poor prognosis, prolonged hospital stays, and poor quality of life especially at advanced disease stages.^5^, ^6^, ^7^ Numerous studies have outlined the importance of nutritional assessment in clinical practice especially in target groups at risk as HF. Importantly, malnutrition is not only common in underweight/lean individuals but is also common in those who are overweight, obese, or even morbidly obese.^8^ Emerging data imply a close link between malnutrition and markers of systemic inflammation generating a hypothesis that increasing adiposity may be protective against the malnutrition–inflammation–cachexia complex which is characteristic for advanced stages of chronic HF.^5^ In recent years, several nutritional screening tools have been advocated to assess nutritional status in HF patients.^8^ The Prognostic Nutritional Index (PNI) provides a simple and objective tool that has been widely used for evaluating nutritional status.^8^, ^9^, ^10^, ^11^

Previous studies investigating the impact of obesity were largely performed in patients with HF with reduced ejection fraction (HFrEF) and with preserved ejection fraction (HFpEF) or in chronic HF regardless of ejection fraction.^2^ The relationship between outcome and BMI in patients with HF and mildly reduced ejection fraction (HFmrEF) is less clear and has rarely been addressed.^12^

Therefore, this study aims (i) to explore the relationship between nutritional status and BMI across the whole spectrum of HF and (ii) to investigate the impact of nutritional status, reflected by PNI, on the obesity paradox across HF phenotypes.

## Materials and methods

### Study population

A single centre, retrospective observational study design has been followed. Patients with chronic HF were enrolled between 2010 and 2020 at the Medical University of Vienna. Detailed study selection criteria have been described before.^13^ Briefly, medical health records and echocardiography database were used to identify patients with HFpEF, HFmrEF, and HFrEF following algorithms that comply with the current guideline diagnostic criteria.^14^ This database includes inpatients and outpatients from the Medical University of Vienna. In accordance with the current guidelines, the following algorithms were applied to identify patients with the respective HF phenotype:

In patients with mildly reduced or preserved left ventricular function (ejection fraction above 40%), at least one of the following criteria were required: structural heart disease as identified by left atrial enlargement and/or left ventricular hypertrophy, or diastolic dysfunction. Moreover, the presence of elevated N‐terminal pro brain‐type natriuretic peptide (NT‐proBNP) values (>125 pg/mL) and symptoms as well as signs of HF were mandatory for study inclusion. HF with a significant reduction in left ventricular systolic function, that is, left ventricular ejection fraction <40%, was designated as HFrEF.

Echocardiographic exams with missing values of interest and patients with primary valve disease were excluded from the study. Likewise, patients without signs and symptoms of HF, with NT‐proBNP values below 125 pg/mL and without measurement of height and weight, were excluded from the analysis. The final study group consisted of 11 995 individuals stratified according to the HF phenotypes.

Targeted keyword search and designated coding from the International Statistical Classification of Diseases (ICD) and Related Health Problems allowed the collection of medical history and laboratory parameters from the electronic local health record database. The process for assigning diagnostic codes is standardized at the Medical University of Vienna. At the time of patient discharge or outpatient presentation, healthcare professionals assign ICD‐codes based on newly diagnosed diseases and the patient's medical history. Routine laboratory parameters were analysed from venous blood samples according to the local laboratory's standard procedure. The study was approved by the institutional ethics review board of the Medical University of Vienna (IRBNr: 2137) with a waiver for informed consent.

### Body mass index

Body mass index was calculated as weight in kilograms divided by height in meters squared (kg/m^2^). Subjects were stratified according to BMI levels <22.5, 22.5–24.9, 25.0–29.9, 30.0–34.9, and ≥35.0 kg/m^2^. To study the relationship between BMI and PNI, the following BMI strata were built: <25, 25–30, >30 and <25, 25–29.9, 30–35, and >35 kg/m^2^.

### Prognostic nutritional index

Albumin levels and total lymphocyte counts were routinely measured during outpatient visits or on admission. The nutritional status was assessed by the PNI according to the following formula: albumin (g/L) + 5 × total lymphocyte count × 10^9^/L. Lower PNI scores indicate worse nutritional status. We used an established cut‐off score of 45 to stratify patients into two groups: low PNI (<45) vs high PNI (≥45).^11^, ^15^, ^16^ As a quality control measure, we investigated the optimal threshold for our study cohort using the Youden index. Concordantly, the optimal threshold value of the PNI score to identify individuals at risk was 46.5.

### Endpoints and follow‐up

The primary endpoint was defined as all‐cause mortality and was assessed via record linkage with the Austrian Death Registry.

### Echocardiographic assessment

Standard transthoracic echocardiograms (2D, Doppler) examinations were performed using commercially available equipment (Vivid E7 and E9, GE Healthcare, Chicago, IL and Acuson S2000, Siemens, Berlin, Germany) according to the current guidelines.^17^ Cardiac morphology was assessed in standard four and two chamber views. Left ventricular systolic function was graded according to ejection fraction cut‐offs. According to the local laboratory standard, left ventricular ejection fraction cut‐offs were ≥50% corresponding for HFpEF, 40–49% for HFmrEF, and <40% for HFrEF. Semiquantitative assessment of right heart function was performed by experienced readers using multiple acoustic windows graded as normal, mild, mild‐to‐moderate, moderate, moderate‐to‐severe, and severe. Valvular regurgitation was quantified using an integrated approach and graded as none, mild, mild‐to‐moderate, moderate, moderate‐to‐severe, and severe. Systolic pulmonary artery pressures were calculated by adding the peak tricuspid regurgitation systolic gradient to the estimated central venous pressure.

### Statistical analysis

Continuous data are expressed as median and interquartile range (IQR) and categorical data as count and percentages. Comparison between groups was performed by the Kruskal–Wallis test, Mann–Whitney *U* test, and the Fisher's exact test as appropriate. Cox proportional hazard regression analysis was applied to assess the impact of BMI and the PNI score on outcome, and the results are presented as hazard ratio (HR) with 95% confidence interval (CI). The covariates presented in *Table* 1 (excluding echocardiographic parameters) with *P*‐values <0.10 in the univariate analysis were considered significant for entry in the multivariate analysis. These variables were age, history of hypertension, diabetes, coronary artery disease, atrial fibrillation, chronic obstructive pulmonary disease, peripheral artery disease, haematocrit, blood urea nitrogen (BUN), NT‐proBNP, cholinesterase, albumin, low density lipoprotein, and lymphocyte count. For multivariate testing of PNI, albumin and lymphocyte count were excluded from the model. To assess whether associations were independent of HF phenotype, the model was additionally adjusted for HF type. Restricted cubic spline curves were generated to illustrate the association of BMI with outcome. Time‐to‐event data are presented as Kaplan–Meier curves. Log‐rank tests were used to compare survival between groups. Additional subgroup analysis was conducted to investigate the impact of BMI on outcome in prespecified subgroups. Two‐sided *P*‐values <0.05 were considered statistically significant. Statistical analysis was performed using SPSS software (IBM SPSS, Chicago, Illinois, USA) version 24 and RStudio (R Foundation for Statistical Computing, Vienna, Austria) version 1.3.1073.

**Table 1:** Baseline characteristics, laboratory, and echocardiographic parameters in the overall cohort and according to heart failure phenotypes

	Overall cohort (*n* = 11 995)	HFpEF (*n* = 6916)	HFmrEF (*n* = 2809)	HFrEF (*n* = 2270)	*P*‐value
Clinical characteristics					
Age, years (IQR)	70 (61–77)	71 (63–78)	70 (60–77)	67 (57–75)	<0.001
Female, *n* (%)	4011 (33)	2826 (41)	660 (23)	525 (23)	<0.001
BMI (IQR)	27.5 (24.5–31.1)	27.7 (24.6–31.5)	27.5 (24.6–30.8)	26.8 (23.9–30.4)	<0.001
<22.5 kg/m^2^, *n* (%)	1328 (11)	725 (10)	275 (10)	328 (14)	0.455
22.5–24.9 kg/m^2^, *n* (%)	2252 (19)	1264 (18)	523 (19)	465 (20)	0.003
25.0–29.9 kg/m^2^, *n* (%)	4640 (39)	2610 (38)	1164 (41)	866 (38)	<0.001
30.0–34.9 kg/m^2^, *n* (%)	2489 (21)	1500 (22)	588 (21)	401 (18)	0.034
≥35 kg/m^2^, *n* (%)	1286 (11)	817 (12)	259 (9)	210 (9)	<0.001
Co‐morbidities					
Hypertension, *n* (%)	7291 (61)	4272 (62)	1762 (63)	1257 (55)	<0.001
Hyperlipidaemia, *n* (%)	4118 (34)	2231 (31)	1107 (39)	780 (34)	<0.001
Diabetes, *n* (%)	3097 (26)	1702 (25)	738 (26)	657 (29)	0.002
Coronary artery disease, *n* (%)	5899 (49)	2825 (41)	1744 (62)	1330 (59)	<0.001
Atrial fibrillation, *n* (%)	3600 (30)	2092 (30)	810 (29)	698 (31)	0.007
Chronic obstructive pulmonary disease, *n* (%)	1590 (13)	898 (13)	353 (13)	339 (15)	<0.001
Peripheral artery disease, *n* (%)	2892 (24)	1656 (24)	695 (25)	541 (24)	<0.001
Laboratory parameters					
Haematocrit, % (IQR)	38 (33–42)	38 (33–42)	38 (33–42)	39 (34–43)	<0.001
Blood urea nitrogen, mg/dL (IQR)	18.4 (14.0–25.5)	17.9 (13.7–24.5)	18.3 (14.0–25.2)	20.7 (15.5–30.2)	<0.001
BChE, kU/I (IQR)	6.5 (5.0–7.9)	6.6 (5.2–8.0)	6.5 (5.1–7.9)	5.9 (4.4–7.4)	<0.001
Albumin, g/L (IQR)	39.2 (35.1–42.4)	39.5 (35.4–42.6)	39.1 (35.0–42.2)	38.5 (34.5–42.1)	<0.001
LDL cholesterol, mg/dL (IQR)	91.6 (67.6–119.4)	93.2 (69.8–121.4)	90.8 (66.2–120.4)	85.8 (63.0–112.0)	<0.001
Total lymphocyte count, ×10^9^/L (IQR)	7.4 (6.0–9.3)	7.3 (5.9–9.0)	7.6 (6.2–9.6)	7.8 (6.4–9.5)	<0.001
NT‐proBNP, pg/mL (IQR)	1128 (405–3163)	749 (321–1893)	1570 (580–3796)	3558 (1529–8088)	<0.001
Echocardiographic parameters					
Left atrial diameter, mm (IQR)	58 (54–64)	58 (54–63)	59 (54–64)	61 (55–67)	<0.001
Left ventricular end‐diastolic diameter, mm (IQR)	47 (43–52)	45 (41–48)	49 (45–54)	57 (51–62)	<0.001
Right atrial diameter, mm (IQR)	57 (52–63)	56 (52–62)	56 (52–62)	58 (51–65)	<0.001
Right ventricular end‐diastolic diameter, mm (IQR)	34 (30–37)	33 (30–37)	34 (30–47)	35 (31–40)	<0.001
Right ventricular function					
Moderately reduced, *n* (%)	1519 (13)	352 (5)	347 (12)	820 (36)	<0.001
Severely reduced, *n* (%)	299 (3)	48 (1)	32 (1)	219 (10)	<0.001
Mitral regurgitation (severe), *n* (%)	1178 (10)	292 (4)	290 (10)	596 (26)	<0.001
Systolic pulmonary artery pressure, mmHg (IQR)	44 (37–54)	43 (36–54)	44 (36–54)	48 (39–59)	<0.001

Continuous variables are given as median and interquartile range (IQR), and counts are given as numbers and percentages (%).

BChE, butyrylcholinesterase; BMI, body mass index; HFmrEF, heart failure with mildly reduced ejection fraction; HFpEF, heart failure with preserved ejection fraction; HFrEF, heart failure with reduced ejection fraction; LDL, low density lipoprotein; NT‐proBNP, N‐terminal pro brain‐type natriuretic peptide.

## Results

### Study population

Of the 11 995 patients enrolled, 6916 (58%) had HFpEF, 2809 (23%) HFmrEF, and 2270 HFrEF (19%). Median age was 70 years (IQR: 61–77), and the majority of patients were men (*n* = 7984, 67%). The most common comorbidity was hypertension with 61% (*n* = 7219). Individuals with HFrEF had significantly, but clinically not meaningful, lower BMI values than the HFmrEF and HFpEF group (26.8 [IQR 23.9–30.4], 27.5 [IQR 24.6–30.8], 27.7 [IQR 24.6–31.5], respectively; *P* = 0.001). *Table* 1 shows the baseline characteristics of the overall cohort and for the respective HF subtypes.

### Impact of body mass index on outcome for the overall cohort and across the heart failure spectrum

**Figure 1:**
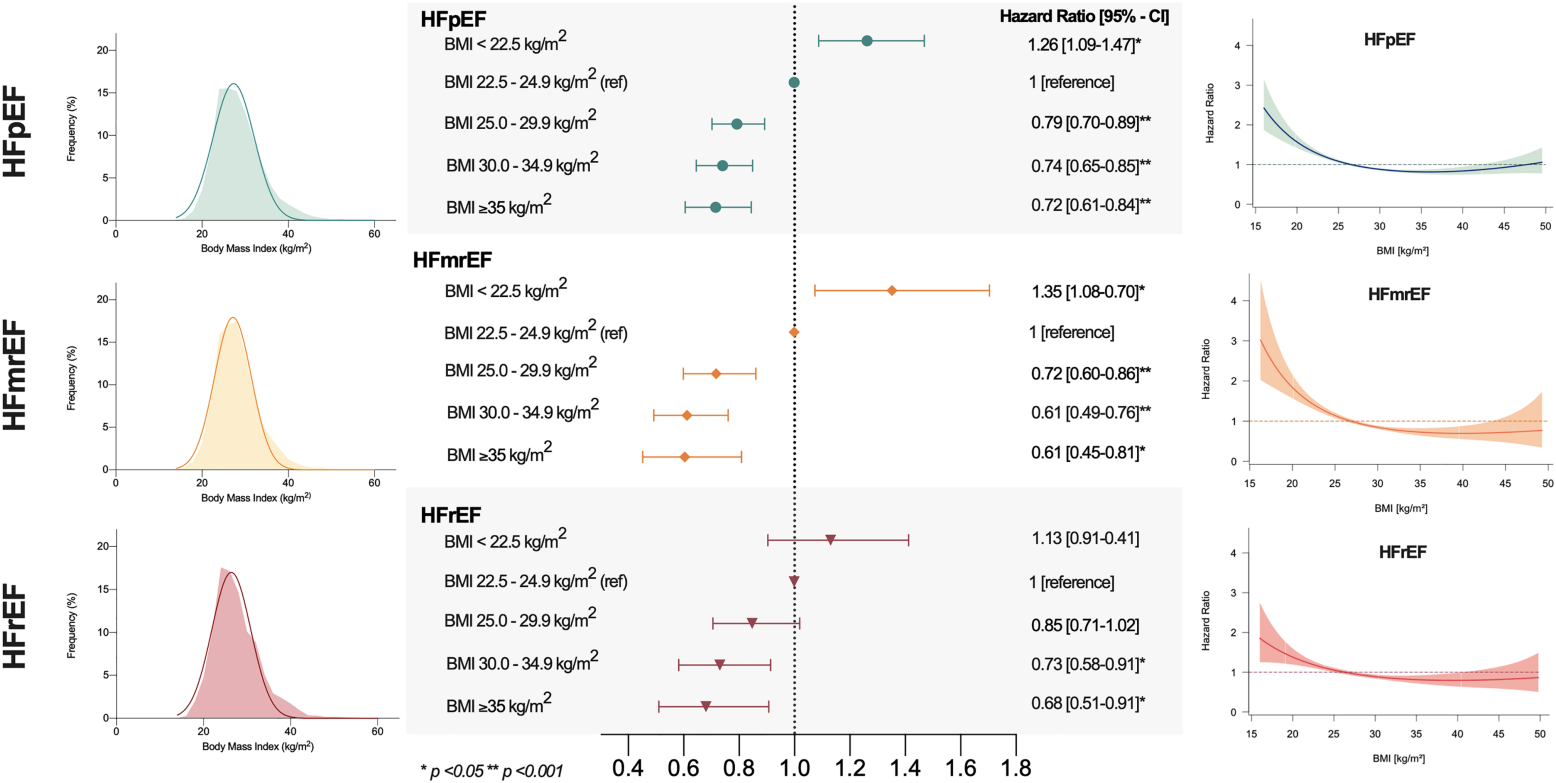
The obesity paradox across the spectrum of heart failure (HF). Distribution of body mass index (BMI) (left), the hazard ratios (HR) for all‐cause mortality with 95% confidence intervals (CI) according to the BMI strata (middle) and restricted spline curves examining the association of BMI and outcome (right) are shown for heart failure with preserved ejection fraction (HFpEF), heart failure with mildly reduced ejection fraction (HFmrEF), and heart failure with reduced ejection fraction (HFrEF).

**Figure 2:**
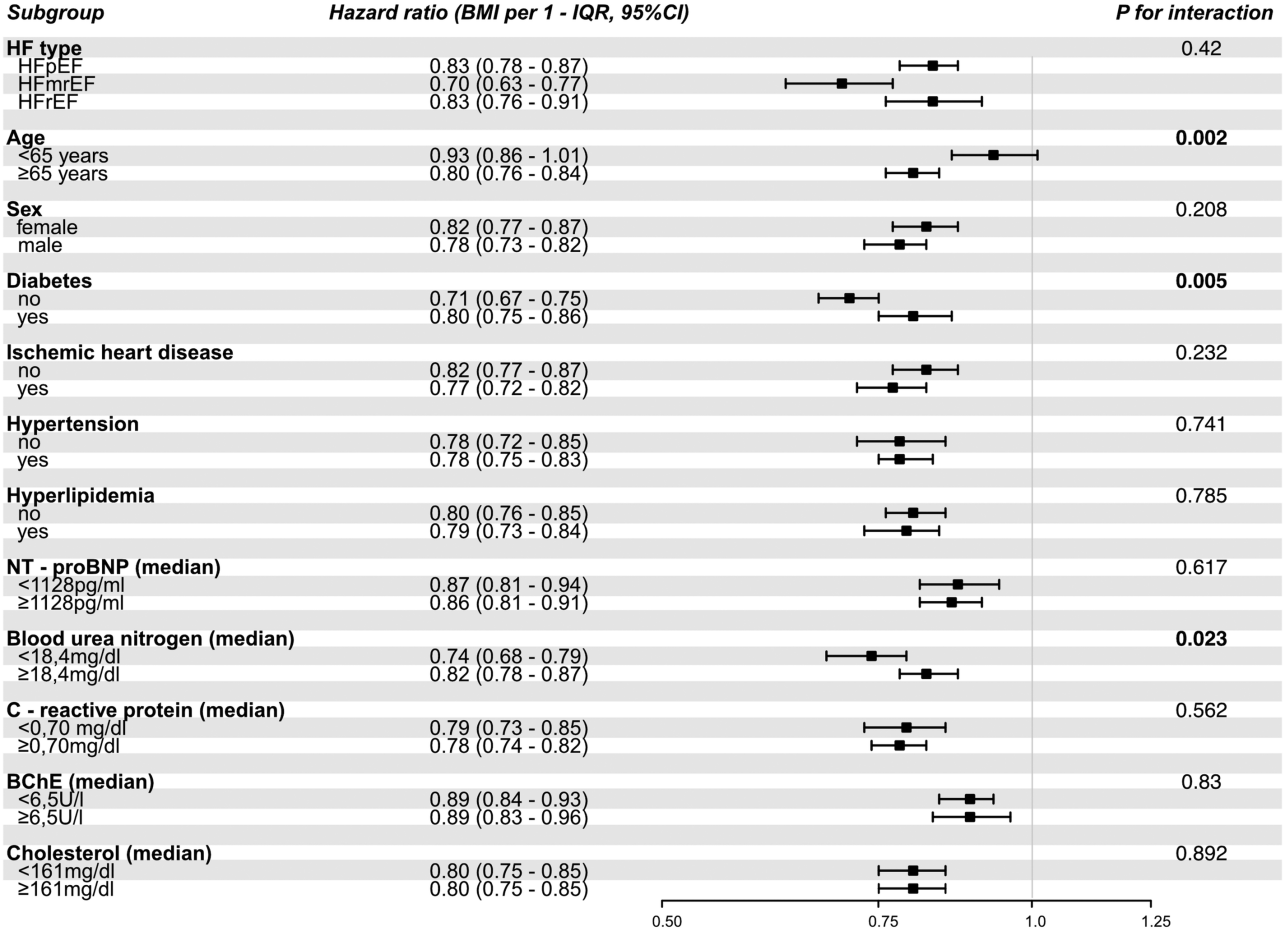
Association of body mass index (BMI) and all‐cause mortality in various subgroups. The median value was used as the cut‐off for continuous data.

During a median follow‐up time of 44 months (IQR 19–76), a total of 3718 (30%) deaths were observed. Our results demonstrate a near U‐shaped association between BMI and mortality for the overall cohort of HF with an inverse relationship in individuals with a BMI < 35 kg/m^2^ (Supporting Information, *Figure* S1). BMI was consistently associated with lower risk for all‐cause mortality in both the HF phenotypes and various subgroups as shown in *Figures* 1 and 2. Univariate Cox regression analysis revealed a uniformly significant inverse association between BMI per IQR and all‐cause mortality for all HF types (HFpEF: HR: 0.83 [CI 0.78–0.87], *P* < 0.001, HFmrEF: HR: 0.70 [CI 0.63–0.77], *P* < 0.001 and HFrEF: HR: 0.83 [0.76–0.91, *P* < 0.001]; *P*‐for‐interaction: 0.420). Significant interactions were observed for BMI and diabetes, kidney function, and age with a more pronounced association in patients without diabetes (no diabetes: HR: 0.71 [0.67–0.75], *P* < 0.001; diabetes: HR: 0.80 [CI 0.75–0.86], *P* < 0.001; *P*‐for‐interaction: 0.005), with lower BUN levels (BUN <18.4 mg/dL: HR: 0.74 [CI 0.68–0.79], *P* < 0.001; BUN ≥18.4 mg/dL: HR: 0.82 [CI 0.78–0.87], *P* < 0.001; *P*‐for‐interaction: 0.023), and in older individuals (age <65 years: HR: 0.93 [CI 0.86–1.01], *P* = 0.117; age ≥65 years: HR: 0.80 [CI 0.76–0.84], *P* < 0.001; *P*‐for‐interaction: 0.002). There was no significant interaction between BMI and sex, ischaemic heart disease, hypertension, inflammation, liver function, and cholesterol metabolism. In multivariate analysis, BMI per IQR was independently associated with better survival (adj. HR: 0.91 [CI 0.86–0.97], *P* = 0.005), and this association remained significant after additional adjustment for HF type (adj. HR: 0.92 [CI 0.86–0.98], *P* = 0.011).

The Kaplan–Meier estimates for the overall survival at 4 years differed significantly between the HF groups with worse survival in the HFrEF population (74.2% for HFpEF, 73.5% for HFmrEF, and 66.4% for HFrEF; log‐rank *P* < 0.001). Cubic spline modelling and the relative hazards for BMI strata and outcome for the respective HF phenotypes are depicted in *Figure* 1. The association of increasing BMI values and favourable outcome was consistent across all HF phenotypes. Compared with patients with BMI levels between 22.5 and 24.9 kg/m^2^ (reference group), patients in the lower BMI category (<22.5 kg/m^2^) had significant worse outcome, while individuals in higher BMI categories (>25 kg/m^2^) had a significant survival advantage independent of HF phenotype.

### Impact of prognostic nutritional index on the obesity paradox

**Figure 3:**
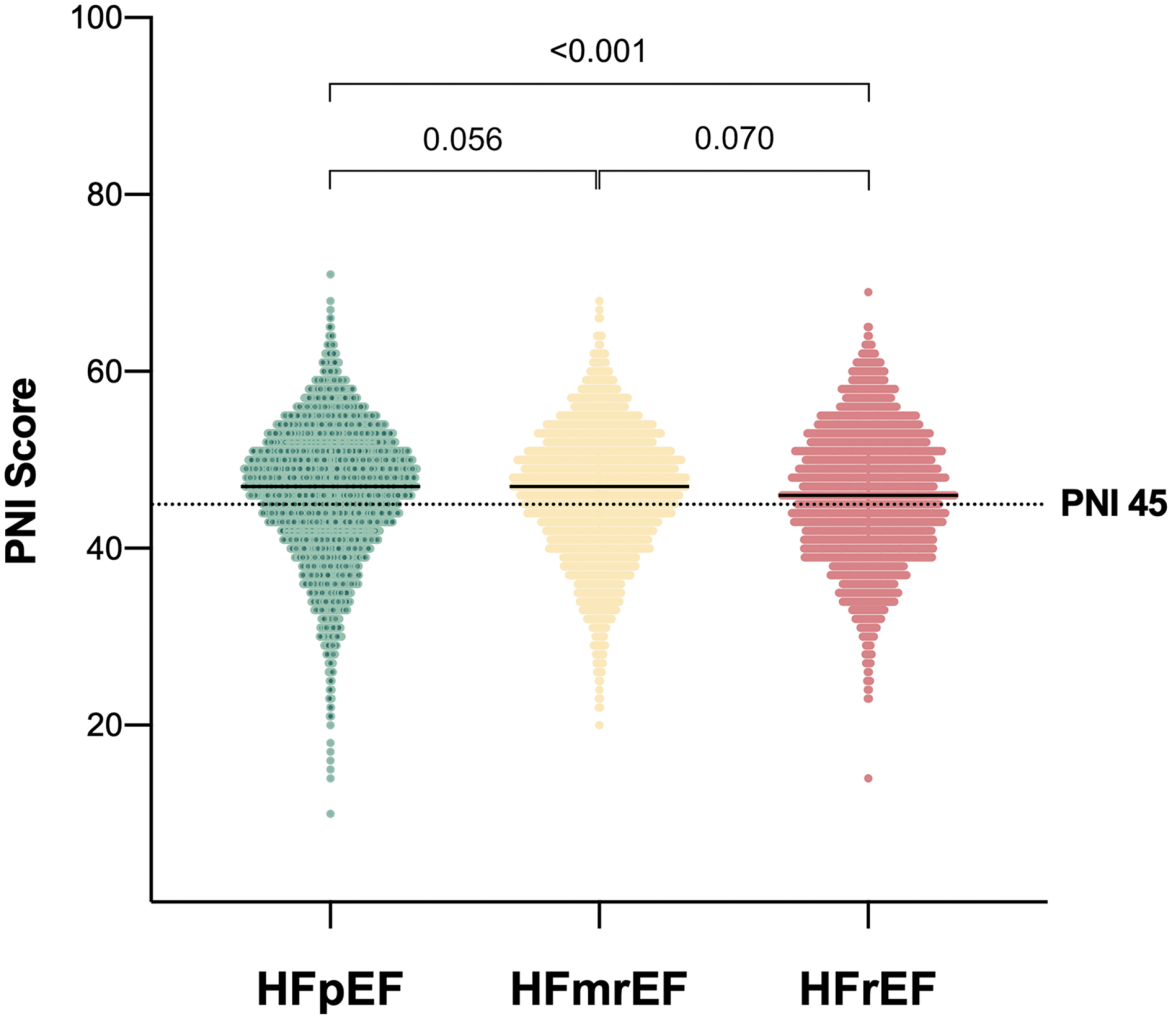
Distribution of the prognostic nutritional index (PNI) according to heart failure phenotype. The dashed vertical line indicates the cut‐off for malnutrition (PNI < 45).

Data for calculation of the PNI score were available in 10 005 patients. Median PNI was 46.9 (IQR 41.8–51.3) in the overall cohort. Patients with HFrEF had significantly lower PNI scores compared with individuals with HFpEF but not HFmrEF (46.0 [IQR 40.8–51.3] vs. 47.2 [IQR 42.2–51.5], *P* < 0.001; vs. 46.8 [IQR 41.8–51.0], *P* = 0.070) (*Figure* 3).

**Figure 4:**
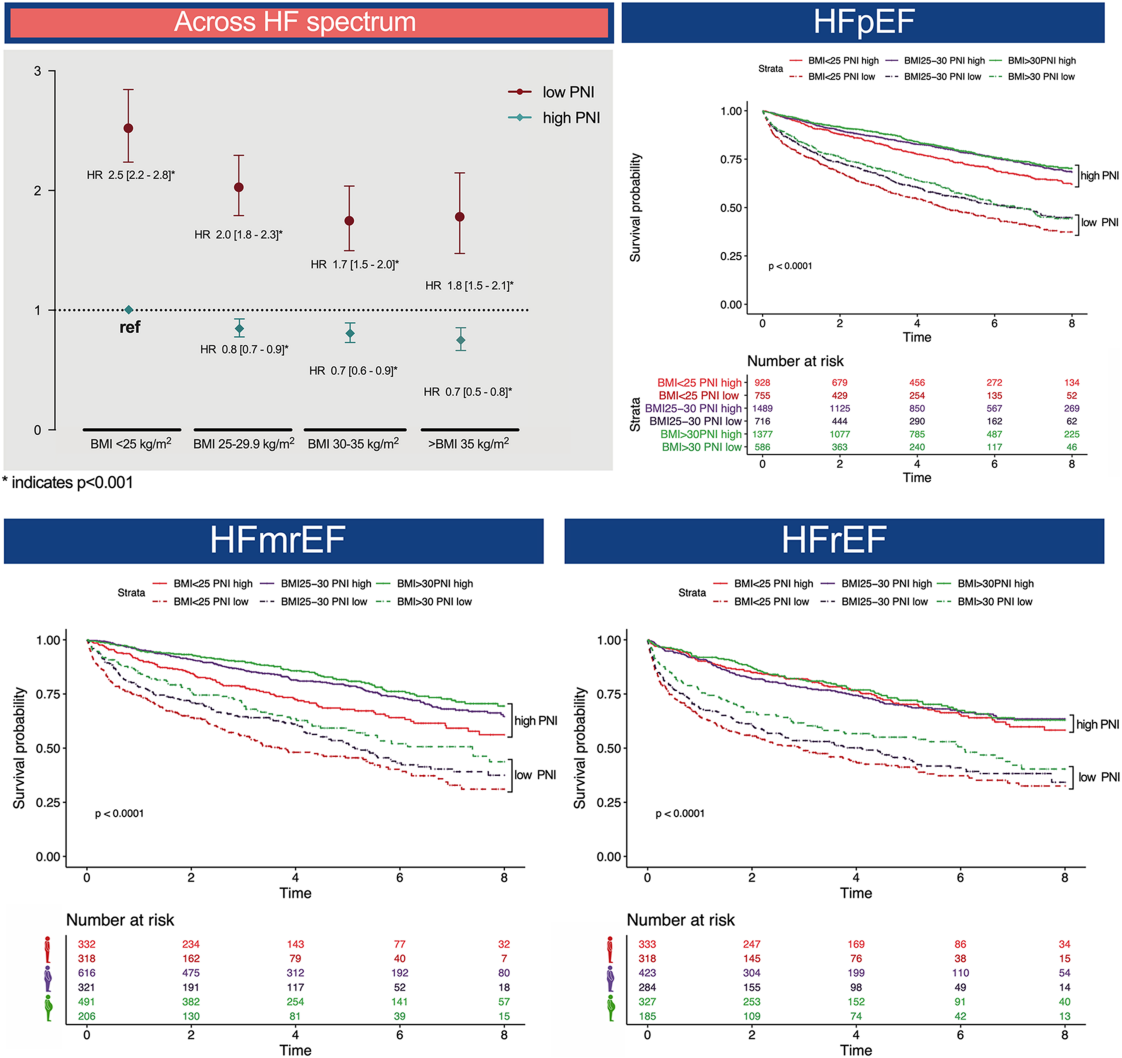
(Left) Hazard ratios (HR) for all‐cause mortality with 95% confidence intervals are shown for body mass index (BMI) in relation to low and high prognostic nutritional index (PNI). (Right) Kaplan–Maier curves showing all‐cause mortality for prespecified BMI groups stratified by nutritional status. The *P*‐value of a log‐rank test for trend is shown for each plot.

In all three HF phenotypes, high PNI was associated with improved survival in the univariate analysis (HFpEF: HR: 0.93 [CI 0.92–0.93], HFmrEF: HR: 0.92 [CI 0.91–0.93], HFrEF: HR: 0.93 [CI 0.92–0.94]; *P* < 0.001 for all). After multivariate adjustment, PNI remained significant to predict outcome (adj. HR: 0.96 [CI 0.95–0.97], *P* < 0.001), additionally adjusting for HF type yielded similar results (adj. HR: 0.96 [CI 0.96–0.97], *P* < 0.001). *Figure* 4 illustrates the relationship between BMI and outcome depending on malnutrition status for the overall cohort and HF phenotypes. Although a higher BMI was associated with generally favourable outcome in both PNI groups separately (Supporting Information, *Table* S1), the hazard for patients with poor nutritional status (low PNI) was 1.8‐ to 2.5‐fold higher compared with the reference group of normal weight high PNI patients. Kaplan–Meier analysis confirmed the survival advantage in obese patients with high PNI, whereas the outcome becomes less favourable with decreasing BMI and especially low PNI for all HF phenotypes.

## Discussion

### Main findings

This study reinforces the evidence of an obesity paradox across HF regardless of sex, ischaemic aetiology of HF and especially HF phenotype with similar findings for HFpEF, HFmrEF, and HFrEF in a comprehensive cohort of HF patients. Our data underscore the importance of nutritional status in relation to the obesity paradox. While increasing BMI is indeed associated with better outcomes in both normal and malnourished patients, obesity alone cannot outbalance malnutrition, with worse prognosis observed in obese but malnourished patients compared with normal weight patients with normal nutritional index.

Whether a low PNI already reflects malnutrition within the scope of a proinflammatory predisposition marking a more advanced state of disease or is a modifiable factor remains to be investigated. The results, however, encourage clinicians to further assess nutritional status in otherwise obese HF patients to identify malnourished individuals with an intrudingly poor prognosis, which seems contra‐intuitive.

### Obesity paradox in heart failure

An inverse association between outcome and BMI has been repeatedly demonstrated in individuals with HFrEF and HFpEF.^2^ To the best of our knowledge, there is only one study reporting the presence of the obesity paradox in HFmrEF with 947 individuals.^12^ With 11 995 patients enrolled (58% HFpEF, 23% HFmrEF, and 19% HFrEF), the current report presents the largest study so far confirming the obesity paradox irrespective of HF phenotype.

The presence of the obesity paradox in HF should clearly not be seen as a promotion of obesity in the general population or individuals without cardiovascular disease; however, recommendations for patients with established disease are not clear. Indeed, assessment of BMI alone may not capture the whole picture of metabolic health as it poorly reflects body composition and metabolic capacity or their trajectories.^18^ Therefore, caution should be exercised when recommending weight loss in patients considering only BMI. A number of hypotheses have been drawn to decipher the inverse association of BMI and outcome. The resilient protection of high BMI in patients with HF may be explained by greater metabolic reserve, reduced sympathetic activity, attenuated response to neuroendocrine stimuli, and less catabolic state.^4^, ^19^ In addition, adipose tissue may attenuate inflammatory responses through synthesis of beneficial adipokines.^20^ The endotoxin/lipid hypothesis suggests that higher circulating lipoproteins in obese patients may enhance endotoxin‐scavenging activity, resulting in lower proinflammatory cytokine production.^21^ In light of these considerations, excess body weight is thought to counteract the catabolic effects of HF and thus provide a metabolic cushion to mitigate disease progression.

However, because data on the obesity paradox mostly emerged from observational studies, bias underlying the paradoxical association such as confounding or reverse causation need to be considered. This wide term includes confounding by pre‐existing weight loss or other predictors of low body weight (e.g. stage and grade of disease, malnutrition, and smoking status) which in turn increase the risk of adverse outcome.^22^ Irrespective of that, one may also argue that lower BMI remains a surrogate for advanced disease stage.

### Malnutrition in heart failure

Malnutrition is common in patients at advanced disease stage and carries poor prognosis.^23^ It is defined as a metabolic state resulting from a chronic imbalance between anabolism and catabolism leading to loss of appetite, malabsorption, inflammation, muscle wasting, and cachexia. In HF, chronic congestion accompanied by gastrointestinal congestion leads to decreased nutrient intake further driving muscle wasting.^23^, ^24^ Disturbed gut perfusion and impaired microcirculation of the intestine results in local oedema, abnormal mucosal permeability, and increased endotoxin absorption further promoting a proinflammatory milieu.^25^ The consequent inflammation is considered to be a key driver of cardiac cachexia representing the hallmark of end‐stage chronic HF.^26^ To date, there are no clear assessment criteria, universally accepted definitions, or standardized methods for determining nutritional status in patients with HF. PNI calculated with serum albumin concentration and total lymphocyte count presents an easy and objective screening tool to detect cardiometabolic derangements in HF that may allow early detection of both malabsorption and inflammatory disturbances. Numerous reports have demonstrated that albumin is a strong predictor for outcome across the spectrum of HF and provides comparable prognostic information to simple or multidimensional malnutrition tools.^27^, ^28^ However, as albumin concentration is known to be affected by several non‐nutritional factors such as hydration state, liver dysfunction, capillary permeability, nephrotic syndrome, infection, and malignancies, the use of albumin alone may not provide a comprehensive and accurate reflection of nutritional status. Lymphocyte count constitutes another determining factor of the PNI score. Nutritional deprivation is commonly associated with impaired immune response leading to lymphocyte depletion.^29^ Previous reports have shown that total lymphocyte count correlates with various established nutritional assessment tools.^,30^ Therefore, combining serum albumin levels and the lymphocyte count to create the PNI may be useful as a screening tool for patients at risk of malnutrition who may benefit from a more detailed nutritional assessment. Earlier reports have shown that low PNI is independently associated with poor outcome in patients with either HFrEF or HFpEF and in chronic HF regardless of ejection fraction.^8^, ^10^, ^11^ To our knowledge, there are no previous studies investigating PNI specifically in patients with HFmrEF. Clearly, our data demonstrate that a substantial proportion of patients with HF, especially HFrEF, are at great risk for malnutrition. Notably, nearly one in two patients with HFrEF had signs of malnutrition. This finding appears consistent with earlier studies reporting a prevalence to be as high as 69% in some HF populations.^9^ Moreover, the present study demonstrates that nutritional status assessed by PNI is an independent predictor of mortality across the spectrum of HF, now embracing also HFmrEF.

### The impact of malnutrition on the obesity paradox

Obese patients in the general population are recommended to lose weight; however, these recommendations become uncertain for individuals with obesity and concomitant HF.^31^ Importantly, BMI alone will not distinguish between metabolically healthy and metabolically unhealthy individuals. Although our data confirm the observation of protective effects of BMI on outcome in individuals with HF, a closer look incorporating signs of malnutrition revealed that increased body weight cannot reverse the negative impact of malnutrition. While obese patients experience a 30% risk reduction, malnutrition at least doubles the risk for death.

Patients with low BMI and poor nutritional status represent a group of patients with diminishing metabolic reserve and with great risk for cardiac cachexia or already established cardiac cachexia. Undoubtedly, this group of patients are at extremely high risk for adverse outcome and should be closely monitored in daily routine practice. The data of this report however also imply that nutritional assessment is also essential in obese patients, because once signs of malnutrition become apparent, the risk of fatal events increases dramatically although obesity is suggestive for better outcomes based on the obesity paradox.

### Limitations

The results of our study should be interpreted in light of its limitations. First, based on the retrospective observational study design, the risk of bias and residual confounding cannot be completely ruled out, although we attempted to adjust for the confounding factors. The observational nature of this report allows us to demonstrate associations, but no inferences can be made about causal relationships. Second, medical history data were derived from the health care provider information system, using codes from the International Statistical Classification of Diseases and Related Health Problems, which could have led to a misclassification and/or underestimation of comorbid conditions. Third, left ventricular ejection fraction could not be measured quantitatively in all patients using biplane Simpson's method, due to the limitations inherent to the method such as poor image quality, dyssynchrony, regional wall motion abnormalities, and foreshortening. Fourth, we did not evaluate measures of frailty, which may be useful to explain the relationship between being underweight and all‐cause mortality especially in the elderly age group.

## Conclusion

The obesity paradox applies for the whole spectrum of HF irrespective of phenotype, that is, HFrEF, HFmrEF, and HFpEF. Nonetheless, the prognosis in obese patients varies greatly depending on the status of malnutrition, which underlines the importance of additional nutritional assessment in lean, but especially in obese patients for individual patient risk stratification.

## Funding

This work was supported by the grant from the Austrian Science Fund (KLI 700‐B30).

## Conflict of interest

The authors declare that they have no relevant conflict of interest.

## Supporting information




**F** **igure S1.** Restricted spline curve showing the association of body mass index and all‐cause mortality in the overall population.
**Table S1.** Hazard ratios for all‐cause mortality with 95% confidence intervals are shown for BMI in relation to low and high PNI.
