# Supplementary material for: Malnutrition outweighs the effect of the obesity paradox
Source: J Cachexia Sarcopenia Muscle. 2022 Mar 29;13(3):1477–86. doi: 10.1002/jcsm.12980 (PMC9178364; doi:10.1002/jcsm.12980)
Supplement: Supplementary file 1 — F igure S1. Restricted spline curve showing the association of body mass index and all‐cause mortality in the overall population. Table S1. Hazard ratios for all‐cause mortality with 95% confidence intervals are shown for BMI in relation to low and high PNI. [file JCSM-13-1477-s001.docx]

**Figure S1.** Restricted spline curve showing the association of body mass index (BMI) and all-cause mortality in the overall population.

|  | PNI low | |  | PNI high | |
| --- | --- | --- | --- | --- | --- |
|  | Crude HR (95% CI) | p-value |  | Crude HR (95% CI) | p-value |
| BMI<25 | reference |  |  | reference |  |
| BMI 25-30 | 0.79 [0.69 to 0.89] | <0.001 |  | 0.82 [0.73 to 0.91] | <0.001 |
| BMI 30-35 | 0.73 [0.63 to 0.85] | <0.001 |  | 0.71 [0.61 to 0.81] | <0.001 |
| BMI >35 | 0.66 [0.54 to 0.80] | <0.001 |  | 0.72 [0.60 to 0.86] | <0.001 |

**Table S1.** Hazard ratios (HR) for all-cause mortality with 95% confidence intervals (CI) are shown for body mass index (BMI) in relation to low and high prognostic nutritional index (PNI).
